# Characteristics of Departments That Provided Primary Support for Households with Complex Care Needs in the Community: A Preliminary Cross-Sectional Study

**DOI:** 10.3390/healthcare9040403

**Published:** 2021-04-01

**Authors:** Kyoko Yoshioka-Maeda, Hitoshi Fujii

**Affiliations:** 1 Department of Health Promotion, National Institute of Public Health, 2-3-6, Minami, Wako-shi, Saitama 351-0197, Japan; 2 Department of Medical Statistics, School of Nursing, Mejiro University, 320 Ukiya, Iwatsuki-ku, Saitama-shi, Saitama 339-8501, Japan; h.fujii@mejiro.ac.jp

**Keywords:** aging, community, complex care needs, economic distress, family characteristics, healthcare delivery, households, mental health, public health, referral and consultation

## Abstract

To prevent emergency admissions and save medical costs, support should be provided to households that include people with complex care needs to allow them to continue living in their own homes. This community-based, cross-sectional study was conducted to (1) identify which departments that public health nurses (PHNs) worked have been the primary providers of support for households with complex care needs and (2) clarify the length of time required by each department to resolve primary health problems. We analyzed 148 households with complex care needs that were registered in City A from April 2018 to July 2019. Four types of departments were the primary support providers for complex care households: the department supporting persons with disabilities (*n* = 54, 36.5%), public/community health centers (*n* = 47, 31.8%), department of older adults (*n* = 29, 19.6%), and welfare offices (*n* = 18, 12.2%). The Mantel–Cox test showed that welfare offices mainly supported households in economic distress and needed significantly less time to resolve their primary health issues than other departments. For early detection and resolution of primary health problems for households with complex care needs, PHNs and healthcare professionals should focus on their economic distress and enhanced multidisciplinary approaches.

## 1. Introduction

To reduce health inequities in the community, it is crucial to address the root causes of inequity—the social determinants of health [1]. In efforts to develop sustainable healthcare systems, supporting community-dwelling residents’ complex care needs, such as multiple chronic health problems and social needs, has been a big challenge for local governments [2], as multiple physical and mental health problems as well as biopsychosocial problems—such as literacy and economic status—affect patients’ complex health conditions [3]. Despite communities providing traditional healthcare services based on the age and needs of residents, it has been difficult to coordinate each service. Thus, households with complex care needs have faced health inequities such as a lack of the multidisciplinary services these cases often need [4,5,6]. Realizing health equity that enables everyone to have an opportunity to reach their best health is crucial for securing social justice and promoting health in the community.

At first, healthcare staff in hospitals felt the burden of caring for people with complex care needs [7]. Thus, previous studies focused on medical costs [3,8] and the average length of admission days for such patients [9], management of chronic diseases, and related medications [10]. These patient-oriented and medical-oriented care complexity factors would relate to the care burden of healthcare staff and time required for discharge.

However, they have not focused on the cases’ life-related factors and the health and welfare service aspects that have supported the patients in continuing their daily lives in community settings. Little is known about which departments have supported people with complex care needs and what kind of problems they addressed [5]. Thus, a community-based study is needed to determine the length of time required for health and welfare departments to resolve the primary health problems of community-dwelling citizens with complex care needs [2]. Additionally, supporting people to continue to live in their own homes and manage their daily lives is a crucial strategy for preventing emergency admissions and saving medical costs in aging societies.

Since Japan has become a super-aged society that has an aging rate of over 25%, there has been a shift from hospital-centered medical care to more community-based home care [11]. The national government launched a community-based integrated care center (CICC) system in 2006 for supporting the lives of older adults in their communities [12]. However, this system only provided individual care for older adults and did not consider the impact of their family members with complex care needs who also have health issues [13]. For example, a CICC serving older adults does not provide support for their children in their 40s and 50s who have social withdrawal and live in the same household [14]. Thus, this community-based home care service providing system based on the age of older adults faces difficulties with supporting households with complex care needs, and that conflicts with the same traditional sectionalism issue.

To overcome the adverse effect of vertically divided care administration, the national government has promoted cross-sectional coordination for the whole household of a person with complex care needs to include their families; each local government has been responsible for coordinating whole-household care in their communities since 2017 [15]. In Japan, PHNs who have national licenses and 60% of whom have worked in local governments, play a pivotal role in coordinating cross-sectional issues, providing individual care, and developing needs-oriented policies in each community [16,17]. However, to the best of our knowledge, little is known about what kind of departments that PHNs have worked in and how they have been supporting people with complex care needs and the average length of service required by each supporting department to resolve their primary health problems [5,18]. Therefore, we sought to promote a multidisciplinary community-level approach for supporting households with complex care needs to allow people to continue living in their own homes, prevent emergency admissions, and reduce medical costs. Thus, the aims of this study were to (1) describe which local departments that PHNs have worked that have supported households with complex care needs and (2) clarify the length of time needed for each department to resolve the primary health problems of these households.

## 2. Materials and Methods

### 2.1. Study Design and Participants

We conducted a cross-sectional study to describe the characteristics of cases involving complex care needs and the departments that provide such care to individuals. Based on previous research [2,13,19], we defined “cases with complex care needs in a community-setting” as those that (1) involve a person with multiple health and life-related issues, including socioeconomic and legal issues, (2) require support from various departments, and (3) have led to healthcare professionals who have supported cases experiencing significant difficulties in their daily practices.

The study participants were those registered in City A as cases requiring complex care to support their ability to continue to live within the community. City A is one of the residential areas in the Tokyo metropolis, with a population of approximately 570,000 people and 320,000 households. At the time of the study, the population aging rate (the percentage of the elderly aged ≥65 years in the population) was 22.1%, and the youth population (under 15 years old) was 10.3%. Since April of 2018, City A has developed a home care and life support center promoting multidisciplinary coordination of the staff of various departments providing individual care and services to persons with complex care needs and their families as whole households [18]. The center has implemented cross-sectional coordination and supervision of each complex case and the care provided by each department’s staff. The staff of each department assessed cases with complex care needs in a community setting to check who met the definition. They consulted the center for support. The staff of the center assessed the urgency and complexities of care needs and the root of the households’ primary health issues. When the director and the department chief of the center identified the urgent cases, they supported each department’s healthcare staff to save the lives of the cases. Only non-urgent cases were registered at the center as the households with complex care needs. The center held care meetings to share information and assessments regarding the households with all relevant staff members, develop care plans for the cases, and share each staff member’s responsibilities. When the center monitored care progress and judged their primary health issues were resolved and no longer needed the support, they ended their support. The director and the department chief of the center collaborated to provide anonymized data of 155 households with complex care needs newly registered at the center from 1 April 2018 to 31 July 2019 [18]. Because of the limited number of the registered cases, we did not conduct a power analysis.

### 2.2. Data Collection and Measures

The center’s staff registered the data of each case using a form provided by the national government for the monthly reporting system required to receive subsidization. PHNs combined this data with their nursing records for each case.

Variables of this study included five categories, such as the department that primarily supported each case and the household type of each case. In the national reporting format, it is defined as the department that primarily provides care for each case. Households were divided into seven types: (1) single person <65 years old, (2) single person ≥65 years old, (3) older adult couple, (4) single mother/father with her/his child(ren), (5) older adult with a single child, (6) multigenerational, and (7) other. Additionally, the center’s staff assessed whether the primary health issues had (or had not) been resolved by the end of July 2019. The nursing records had data regarding the date of starting and ending of the support by the center. The director and department chief of the center judged as the resolve of the households’ primary health issue with complex care needed when they did not need support from the center’s staff. The length of time needed to resolve the case’s primary health problem was calculated using the nursing records for each case. Health and life-related issues for each case were the 22 items recorded by the staff on the national government’s monthly reporting form: six health issues, four life issues, four socioeconomic issues, eight family-related issues.

### 2.3. Statistical Analysis

For data analysis, we determined which department had been the primary source of support for each case. Based on the records from each department, we analyzed what kind of health and life-related issues overlapped in each case. Using a survival analysis method, we determined whether there was a difference in the length of time it took each department to resolve each type of the household’s primary health issue. For our purposes in this study, “death” in the survival time analysis was the solution of the problem, and “survival” was the continuation of the problem. The difference between the two groups was analyzed using the Mantel–Cox test, and the *p*-value was Bonferroni corrected.

Statistical analysis was conducted using SPSS for Windows (version 25; IBM Corp, Armonk, NY, USA). *p*-values of less than 0.05 indicated statistical significance.

### 2.4. Ethical Approval

This study protocol was approved by the Institutional Review Board (approval no. NIPH-IBRA#12260) in 2019. The directors of the center and the legal department of City A shared the data of this study. The study complied with the principles of the Declaration of Helsinki.

## 3. Results

### 3.1. Sample Characteristics

After eliminating 7 cases with missing data, we analyzed 148 households with complex care needs. One of four departments had provided the initial support for each household—the department supporting persons with disabilities (*n* = 54, 36.5%), public/community health centers (*n* = 47, 31.8%), the department of older adults (*n* = 29, 19.6%), and the welfare offices (*n* = 18, 12.2%). Table 1 shows the type of households and whether or not the primary health issues had been resolved by the pertinent department by the end of July 2019. An older adult with a single child was the most common type of household (*n* = 44, 29.7%); the second was a single person younger than 65 years old (*n* = 43, 21.9%). Additionally, 71.6% of households’ primary health issues had not been solved by the end of July 2019.

### 3.2. Overlapping Health and Life-Related Issues of Households Primarily Supported by Each Department

There were health and life-related issues that overlapped within each household (Table 2). The public/community health centers had a higher proportion of mental illness than other departments. The department supporting persons with disabilities and the department of older adults was the second most common. In contrast, the older adults’ department had a higher proportion of illness/injury, abuse, and deteriorating family relationships than other departments. The welfare offices supported economic distress more often than other departments did.

### 3.3. The Length of the Care Period Needed to Resolve the Primary Health Issue of the Households Supported Mainly by Each Department

We conducted the Mantel–Cox test to analyze the difference in the length of the care period needed to resolve the primary health issue of the households mainly supported by each department (Table 3). The results showed that the welfare offices needed less time to resolve primary health issues than other departments (Estimate = 135.5, 95% confidence interval = 74.20, 196.86, *p* < 0.001).

## 4. Discussion

This cross-sectional study revealed which departments were the main source of support to households with complex care needs and the length of time the departments needed to resolve their primary health problems. The results show that the department supporting persons with disabilities and the public/community health centers each accounted for more than 30% of the total as those that primarily supported households with complex care needs, especially households with a single person less than 65 years old living alone. Owing to the super-aged society, health services for older people have developed rapidly in Japan [11]. The long-term care insurance system focused on reducing family caregiving and sharing long-term care costs by providing integrated medical and welfare care since 2000 [20]. Despite the national universal health insurance coverage system instituted in 1961, preventive care systems based on self-care and mutual aid along with the welfare system still assume family caregiving is provided in households in Japan [21]. Additionally, Japan’s healthcare systems have not adequately covered differences in age, employment status, or family situation [22]. Thus, the development of health care services for a single person less than 65 years old has been relatively delayed compared to services for older adults in Japan. To help older adults continue living within their communities, PHNs and healthcare professionals should engage in an enhanced multidisciplinary approach to fill the gap in the healthcare coverage for single people less than 65 years old. The enhanced approach should be developed in each community.

We found that the welfare offices which supported economic distress needed less time to resolve primary health issues than other departments. Previous studies focused on vulnerable groups such as persons with mental diseases and homeless people who have experienced economic difficulties and social deprivation [23,24,25], and the importance of cross-sector collaboration for supporting them [26]. Socioeconomic difficulties have exacerbated health conditions of all types in the community [27,28]. To provide effective person-centered care, assessing socioeconomic status regarding health inequities is crucial for understanding the complex care needs of each case [29]. The related economic issues are among the social determinants of health with roots in health inequities [30]. Thus, approaching economic difficulties is key to preventing community-dwellers’ health problems [31,32]. In facts, welfare benefits and a re-employment support system would improve mental health issues and health inequities [33]. The Japanese government published guidelines for public health nursing practice and requires PHNs to support community people in need regardless of socioeconomic status [17]. However, undergraduate students have fewer opportunities to acquire this assessment skill [34]. Additionally, less attention is given to socioeconomic data than to health conditions in each of the complex cases we examined each community [2]. For improving health equities in the community, PHNs and healthcare professionals should focus more on the economic distress of the cases with complex care needs for earlier resolution of their health and life-related issues. Early resolution of economic distress issues would allow PHNs and healthcare professionals to provide more effective and efficient care for households with complex care needs.

In contrast, other departments that supported overlapping cases of mental illness, abuse, and deteriorating family relationships needed more time to resolve the household’s primary health issues than the welfare office did. Family dysfunction affected the physical and mental health of family members [35] and relates to violence and abuse [36,37]. Additionally, because of the Japanese culture of shame, the family caregiver tended to cover for the shortage of social resources regarding mental illness in each community and tried to manage their health and life-related issues without seeking outside help until they reached their limits [14]. Furthermore, stigma against psychiatric illness has affected help-seeking activities and access to mental health care services [38]. In Japan, deinstitutionalization has been promoted since 2004 to reduce the excessive number of psychiatric beds [39]. Despite the nation-wide survey that showed that Japanese people believed that mental illness could be curable [40], the stigma around mental illness is still relatively strong in Japan [38,40]. Japanese people often have shown negative attitudes toward developing public facilities for persons with mental illness in their community [41]. Thus, social resources regarding mental health are still insufficient in Japan [14]. The results suggest that departments that supported people with mental illness would be an initial consultation entrance for them and their families. Additionally, family dysfunction and the delay of help-seeking affect and prolong the resolution of health and life-related issues. For early detection and resolution of primary health problems for complex cases, PHNs, and healthcare professionals should identify overlapping family dysfunction with mental health or abuse and develop needs-oriented services regarding mental health to allow cases to continue living within the community rather than in long-term care.

### Limitations and Future Research

There are some limitations in this study. As the study sample was small and limited, we could not conduct a power analysis. The varying backgrounds of healthcare staff could affect the time needed to resolve clients’ primary health and life-related issues. Due to data collection using the same format as the national government does for subsidization, the generalization of the results may be limited to other municipalities in Japan. The educational background data for each household were not provided by the support center, and we could not validate this format. Future research should collect these data to reveal the causal relationship between complex cases and their health and life-related issues in order to identify the most suitable type of support to address those issues. Additionally, PHNs and healthcare staff should assess the economic distress at the beginning of households’ support with complex care needs. The development of an assessment scale for identifying care needs of the households with complex care needs remains necessary. Furthermore, PHNs and healthcare staff should develop a new multidisciplinary healthcare system, especially for single people less than 65 years old that fills the gap in the existing healthcare systems that are needed in each community.

## 5. Conclusions

In this cross-sectional study, welfare offices primarily supported households in economic distress and needed significantly less time to resolve cases’ primary health issues than other departments did. The results suggest that, for early detection and resolution of primary health problems for households with complex care needs, PHNs and healthcare professionals should focus on their economic distress and develop a new multidisciplinary healthcare system that fills the gaps in existing healthcare systems to enhance health equities in the community.

## Figures and Tables

**Table 1:** Demographic data of the households supported by each department.

Variables	Total	Department Supporting Persons with Disabilities	Public/Community Health Centers	Department of Older Adults	Welfare Offices
	(*N* = 148) (%)	(*n* = 54)	(*n* = 47) (%)	(*n* = 29) (%)	(*n* = 18) (%)
Type of households					
A single person <65 years old	43 (29.1)	19 (44.2)	19 (44.2)	0 (0.0)	5 (11.6)
A single person ≥65 years old	15 (10.1)	0 (0.0)	4 (26.7)	4 (26.7)	7 (46.7)
Older adult couple	4 (2.7)	0 (0.0)	1 (25.0)	2 (50.0)	1 (25.0)
A single mother/father with her/his child(ren)	14 (9.5)	7 (50.0)	3 (21.4)	1 (7.1)	3 (21.4)
Older adult person with a single child	44 (29.7)	18 (40.9)	10 (22.7)	15 (34.1)	1 (2.3)
Multigenerational households	4 (2.7)	0 (0.0)	0 (0.0)	4 (100.0)	0 (0.0)
Other	24 (16.2)	10 (41.7)	10 (41.7)	3 (12.5)	1 (4.2)
Whether the primary health issues had not been resolved by the end of July 2019					
Resolved	42 (28.4)	20 (47.6)	2 (4.8)	6 (14.3)	14 (33.3)
Unresolved	106 (71.6)	34 (32.1)	45 (42.5)	23 (21.7)	4 (3.8)

**Table 2:** The proportion of overlapping health and living-related issues of households supported by each department.

Categories	Items	Total	Department Supporting Persons with Disabilities	Public/Community Health Centers	Department of Older Adults	Welfare Offices
Health issues	Illness/Injury	29 (19.6)	7 (13.0)	3 (6.4)	15 (51.7)	4 (22.2)
	Physical disability	18 (12.2)	9 (16.7)	0 (0.0)	6 (20.7)	3 (16.7)
	Intellectual disability	33 (22.3)	21 (38.9)	2 (4.3)	7 (24.1)	3 (16.7)
	Mental illness	108 (73.0)	37 (68.5)	46 (97.9)	15 (51.7)	10 (55.6)
	Dementia	19 (12.8)	4 (7.4)	3 (6.4)	10 (34.5)	2 (11.1)
	Addiction	16 (10.8)	1 (1.9)	5 (10.6)	6 (20.7)	4 (22.2)
Life-related issues	Unsanitary; trash in the house	14 (9.5)	4 (7.4)	5 (10.6)	3 (10.3)	2 (11.1)
	Neighborhood trouble	21 (14.2)	7 (13.0)	8 (17.0)	3 (10.3)	3 (16.7)
	Social isolation	2 (1.4)	1 (1.9)	1 (2.1)	0 (0.0)	0 (0.0)
	Refusing the support	3 (2.0)	3 (5.6)	0 (0.0)	0 (0.0)	0 (0.0)
Economic-related issues	Unemployment	3 (2.0)	0 (0.0)	3 (6.4)	0 (0.0)	0 (0.0)
	Unstable work	9 (6.1)	3 (5.6)	2 (4.3)	4 (13.8)	0 (0.0)
	Multiple debts	6 (4.1)	3 (5.6)	0 (0.0)	3 (10.3)	0 (0.0)
	Economic distress	36 (24.3)	7 (13.0)	10 (21.3)	8 (27.6)	11 (61.1)
Family-related issues	Abuse	26 (17.6)	8 (14.8)	1 (2.1)	15 (51.7)	2 (11.1)
	Difficulties raising children at home	7 (4.7)	1 (1.9)	2 (4.3)	3 (10.3)	1 (5.6)
	School absenteeism	2 (1.4)	0 (0.0)	1(2.1)	0 (0.0)	1 (5.6)
	Withdrawal	13 (8.8)	1 (1.9)	4 (8.5)	6 (20.7)	2 (11.1)
	Unemployment of his/her child	15 (10.1)	6 (11.1)	3 (6.4)	5 (17.2)	1 (5.6)
	Domestic violence	22 (14.9)	5 (9.3)	8 (17.0)	7 (24.1)	2 (11.1)
	Deteriorating family relationships	23 (15.5)	8 (14.8)	3 (6.4)	9 (31.0)	3 (16.7)
	Difficulties in home care	18 (12.2)	6 (11.1)	3 (6.4)	4 (13.8)	5 (27.8)

Notes: *n* = 148.

**Table 3:** The length of the care period needed in each department to resolve the primary health issue of the households.

Variables	*n*	Estimate	SE	95% CI	*p*-Value
Department Supporting Persons with Disabilities	54	309.4	23.3	(263.80–355.00)	<0.001
Public/Community Health Centers	47	268.9	16.2	(237.25–300.64)	<0.001
Department of Older Adults	29	373.4	27.7	(319.05–427.77)	<0.001
Welfare Offices (reference)	18	135.5	31.3	(74.20–196.86)	-
Total	148	301.7	17.5	(267.27–336.07)	

Notes: *n* = 148; SE: standard error; 95% CI: 95% confidence interval; Mantel–Cox test: chi-square = 37.69 (df = 3), *p* < 0.001; the length of care was measured in days.

## Data Availability

To protect the privacy of research participants, the data of this study are not available to share with other researchers.

## References

[1] World Health Organization (2010). Conceptual Framework for Action on the Social Determinants of Health. https://www.who.int/sdhconference/resources/ConceptualframeworkforactiononSDH_eng.pdf.

[2] McGregor J., Mercer S.W., Harris F.M. (2018). Health benefits of primary care social work for adults with complex health and social needs: A systematic review. Health Soc. Care Community.

[3] Hochman M., Asch S.M. (2017). Disruptive Models in Primary Care: Caring for High-Needs, High-Cost Populations. J. Gen. Intern. Med..

[4] Napoles T.M., Burke N.J., Shim J.K., Davis E., Moskowitz D., Yen I.H. (2017). Assessing Patient Activation among High-Need, High-Cost Patients in Urban Safety Net Care Settings. J. Urban Health..

[5] Rantala R., Bortz M., Armada F. (2014). Intersectoral action: Local governments promoting health. Health Promot. Int..

[6] DeSalvo K.B., Wang Y.C., Harris A., Auerbach J., Koo D., O’Carroll P. (2017). Public Health 3.0: A Call to Action for Public Health to Meet the Challenges of the 21st Century. Prev. Chronic. Dis..

[7] Yoshida S., Matsushima M., Wakabayashi H., Mutai R., Sugiyama Y., Yodoshi T., Horiguchi R., Watanabe T., Fujinuma Y. (2019). Correlation of patient complexity with the burden for health-related professions, and differences in the burden between the professions at a Japanese regional hospital: A prospective cohort study. BMJ Open.

[8] Ritchie C., Andersen R., Eng J., Garrigues S.K., Intinarelli G., Kao H., Kawahara S., Patel K., Sapiro L., Thibault A. (2016). Implementation of an Interdisciplinary, Team-Based Complex Care Support Health Care Model at an Academic Medical Center: Impact on Health Care Utilization and Quality of Life. PLoS ONE.

[9] Shukor A.R., Edelman S., Brown D., Rivard C. (2018). Developing Community-Based Primary Health Care for Complex and Vulnerable Populations in the Vancouver Coastal Health Region: HealthConnection Clinic. Perm. J..

[10] Smith T., Patel T., Akpan A., Clegg A., Watkins D.C., Lightbody C.E., Chauhan U. (2020). A scoping review of community holistic interventions for older people with multimorbidity. Br. J. Gen. Pract..

[11] Arai H., Ouchi Y., Toba K., Endo T., Shimokado K., Tsubota K., Matsuo S., Mori H., Yumura W., Yokode M. (2015). Japan as the front-runner of super-aged societies: Perspectives from medicine and medical care in Japan. Geriatr. Gerontol. Int..

[12] Ministry of Health, Labour, and Welfare (2016). Long-Term Care Insurance System of Japan. https://www.mhlw.go.jp/english/policy/care-welfare/care-welfare-elderly/dl/ltcisj_e.pdf.

[13] Yoshioka-Maeda K. (2019). A comprehensive review of related literature focusing on the elderly persons with difficulty of support in the community comprehensive care center. J. Jpn. Acad. Community Health Nurs..

[14] Yoshioka-Maeda K. (2020). The ’8050 issue’ of social withdrawal and poverty in Japan’s super-aged society. J. Adv. Nurs..

[15] Ministry of Health, Labour, and Welfare (2017). Chiiki Kyousei Shakai no Jitsugen ni Muketa Chiiki Fukushi no Suishin ni Tsuite. https://www.mhlw.go.jp/file/06-Seisakujouhou-12600000-Seisakutoukatsukan/0000189728.pdf.

[16] Yoshioka-Maeda K. (2020). Promoting needs-oriented health programme planning through public health nurses in Japan. J. Adv. Nurs..

[17] Japan Ministry of Health, Labour, and Welfare (2013). Chiiki ni Okeru Hokenshi no Hoken Katudou ni Tuite. http://www.nacphn.jp/topics/pdf/2013_shishin.pdf.

[18] Yoshioka-Maeda K., Fujii H. (2020). Household characteristics of persons with complex care needs in the community: A preliminary study. Nurs. Open.

[19] Buja A., Claus M., Perin L., Rivera M., Corti M.C., Avossa F., Schievano E., Rigon S., Toffanin R., Baldo V. (2018). Multimorbidity patterns in high-need, high-cost elderly patients. PLoS ONE.

[20] Yamada M., Arai H. (2020). Long-Term Care System in Japan. Ann. Geriatr. Med. Res..

[21] Sudo K., Kobayashi J., Noda S., Fukuda Y., Takahashi K. (2018). Japan’s healthcare policy for the elderly through the concepts of self-help (Ji-jo), mutual aid (Go-jo), social solidarity care (Kyo-jo), and governmental care (Ko-jo). Biosci. Trends.

[22] Health Care 2035 Advisory Panel (2015). The Japan Vision: Health Care 2035. https://www.mhlw.go.jp/seisakunitsuite/bunya/hokabunya/shakaihoshou/hokeniryou2035/assets/file/healthcare2035_proposal_150703_summary_en.pdf.

[23] Pottie K., Kendall C.E., Aubry T., Magwood O., Andermann A., Salvalaggio G., Ponka D., Bloch G., Brcic V., Agbata E. (2020). Clinical guideline for homeless and vulnerably housed people, and people with lived homelessness experience. CMAJ.

[24] Dziechciaz M., Filip R. (2014). Biological psychological and social determinants of old age: Bio-psycho-social aspects of human aging. Ann. Agric. Environ. Med..

[25] Staiger T., Waldmann T., Rusch N., Krumm S. (2017). Barriers and facilitators of help-seeking among unemployed persons with mental health problems: A qualitative study. BMC Health Serv. Res..

[26] Tung E.L., Gunter K.E., Bergeron N.Q., Lindau S.T., Chin M.H., Peek M.E. (2018). Cross-Sector Collaboration in the High-Poverty Setting: Qualitative Results from a Community-Based Diabetes Intervention. Health Serv. Res..

[27] Loeb D.F., Binswanger I.A., Candrian C., Bayliss E.A. (2015). Primary care physician insights into a typology of the complex patient in primary care. Ann. Fam. Med..

[28] Gobeil-Lavoie A.P., Chouinard M.C., Danish A., Hudon C. (2019). Characteristics of self-management among patients with complex health needs: A thematic analysis review. BMJ Open.

[29] Webster F., Rice K., Bhattacharyya O., Katz J., Oosenbrug E., Upshur R. (2019). The mismeasurement of complexity: Provider narratives of patients with complex needs in primary care settings. Int. J. Equity Health.

[30] Marmot M. (2017). Social justice, epidemiology and health inequalities. Eur. J. Epidemiol..

[31] Barnes M.C., Donovan J.L., Wilson C., Chatwin J., Davies R., Potokar J., Kapur N., Hawton K., O’Connor R., Gunnell D. (2017). Seeking help in times of economic hardship: Access, experiences of services and unmet need. BMC Psychiatry.

[32] Naik Y., Baker P., Ismail S.A., Tillmann T., Bash K., Quantz D., Hillier-Brown F., Jayatunga W., Kelly G., Black M. (2019). Going upstream—An umbrella review of the macroeconomic determinants of health and health inequalities. BMC Public Health.

[33] Hillier-Brown F., Thomson K., McGowan V., Cairns J., Eikemo T.A., Gil-Gonzale D., Bambra C. (2019). The effects of social protection policies on health inequalities: Evidence from systematic reviews. Scand. J. Public Health.

[34] The Japan Association of Public Health Nurse Educational Institutions Hokenshi Kyouiku Minimum Requirements 2014. The Japan Association of Public Health Nurse Educational Institutions Version. http://www.zenhokyo.jp/work/doc/h26-iinkai-hokenshi-mr-houkoku.pdf.

[35] Repetti R.L., Taylor S.E., Seeman T.E. (2002). Risky families: Family social environments and the mental and physical health of offspring. Psychol. Bull..

[36] Martins R., Neto M.J., Andrade A., Albuquerque C. (2014). Abuse and maltreatment in the elderly. Atención Primaria.

[37] Kageyama M., Solomon P., Yokoyama K., Nakamura Y., Kobayashi S., Fujii C. (2018). Violence Towards Family Caregivers by Their Relative with Schizophrenia in Japan. Psychiatr. Q..

[38] Ando S., Yamaguchi S., Aoki Y., Thornicroft G. (2013). Review of mental-health-related stigma in Japan. Psychiatry Clin. Neurosci..

[39] Organisation for Economic Co-Operation and Development (2014). Making Mental Health Count. https://www.oecd.org/els/health-systems/Focus-on-Health-Making-Mental-Health-Count.pdf.

[40] Kasahara-Kiritani M., Matoba T., Kikuzawa S., Sakano J., Sugiyama K., Yamaki C., Mochizuki M., Yamazaki Y. (2018). Public perceptions toward mental illness in Japan. Asian J. Psychiatr..

[41] Zhang Z., Sun K., Jatchavala C., Koh J., Chia Y., Bose J., Li Z., Tan W., Wang S., Chu W. (2019). Overview of Stigma against Psychiatric Illnesses and Advancements of Anti-Stigma Activities in Six Asian Societies. Int. J. Environ. Res. Public Health.

